# {2-[(2-Bromo-5-meth­oxy­benzyl­idene)amino]-4,5,6,7-tetra­hydro­benzo[*b*]thiophen-3-yl}(phen­yl)methanone

**DOI:** 10.1107/S1600536814008290

**Published:** 2014-04-18

**Authors:** Manpreet Kaur, Jerry P. Jasinski, Thammarse S. Yamuna, H. S. Yathirajan, K. Byrappa

**Affiliations:** a Department of Studies in Chemistry, University of Mysore, Manasagangotri, Mysore 570 006, India; b Department of Chemistry, Keene State College, 229 Main Street, Keene, NH 03435-2001, USA; c Materials Science Center, University of Mysore, Vijyana Bhavan Building, Manasagangothri, Mysore 570 006, India

## Abstract

In the title compound, C_23_H_20_BrNO_2_S, disorder was modeled for the outer two C atoms of the cyclo­hexene ring over two sets of sites with an occupancy ratio of 0.580 (11):0.420 (11). Both rings have a half-chair conformation. The dihedral angles between the mean plane of the thio­phene ring and the benzene and phenyl rings are 9.2 (2) and 66.1 (2)°, respectively. The benzene and phenyl rings are inclined to each other by 74.8 (8)°. In the crystal, mol­ecules are linked by pairs of C—H⋯O hydrogen bonds, forming inversion dimers.

## Related literature   

For applications of 2-amino­thio­phene derivatives, see: Sabnis *et al.* (1999 ▶); Puterová *et al.* (2010 ▶). For the biological and industrial importance of Schiff bases, see: Desai *et al.* (2001 ▶); Karia & Parsania (1999 ▶); Samadhiya & Halve (2001 ▶); Singh & Dash (1988 ▶); Aydogan *et al.* (2001 ▶); Taggi *et al.* (2002 ▶). For a related structure, see: Kubicki *et al.* (2012 ▶). For puckering parameters, see: Cremer & Pople (1975 ▶). For standard bond lengths, see: Allen *et al.* (1987 ▶).
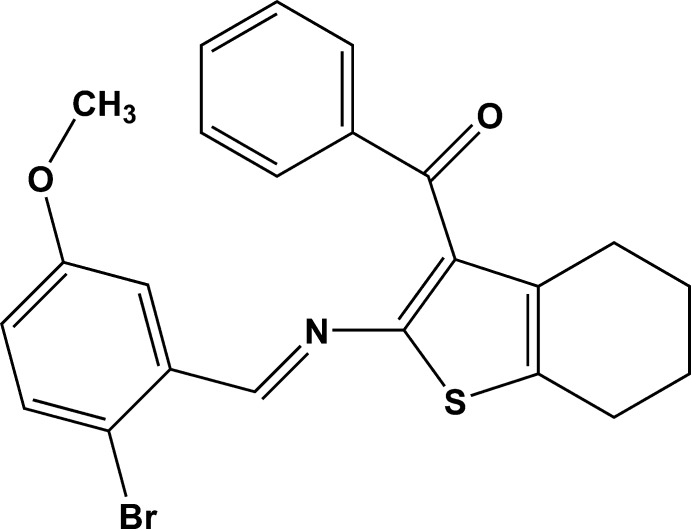


## Experimental   

### 

#### Crystal data  

C_23_H_20_BrNO_2_S
*M* *_r_* = 454.37
Monoclinic, 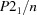

*a* = 8.84813 (17) Å
*b* = 12.5563 (2) Å
*c* = 18.4384 (4) Å
β = 102.363 (2)°
*V* = 2001.00 (7) Å^3^
*Z* = 4
Cu *K*α radiation
μ = 3.92 mm^−1^
*T* = 173 K
0.26 × 0.22 × 0.14 mm

#### Data collection  

Agilent Eos Gemini diffractometer
Absorption correction: multi-scan (*CrysAlis PRO* and *CrysAlis RED*; Agilent, 2012 ▶) *T*_min_ = 0.725, *T*_max_ = 1.000
12404 measured reflections
3853 independent reflections
3440 reflections with *I* > 2σ(*I*)
*R*_int_ = 0.034

#### Refinement  

*R*[*F* ^2^ > 2σ(*F* ^2^)] = 0.032
*wR*(*F* ^2^) = 0.087
*S* = 1.05
3853 reflections
273 parameters
H-atom parameters constrained
Δρ_max_ = 0.56 e Å^−3^
Δρ_min_ = −0.31 e Å^−3^

### 

Data collection: *CrysAlis PRO* (Agilent, 2012 ▶); cell refinement: *CrysAlis PRO*; data reduction: *CrysAlis RED* (Agilent, 2012 ▶); program(s) used to solve structure: *SUPERFLIP* (Palatinus & Chapuis, 2007 ▶); program(s) used to refine structure: *SHELXL2012* (Sheldrick, 2008 ▶); molecular graphics: *PLATON* (Spek, 2009 ▶) and *Mercury* (Macrae *et al.*, 2008 ▶); software used to prepare material for publication: *OLEX2* (Dolomanov *et al.*, 2009 ▶) and *PLATON*.

## Supplementary Material

Crystal structure: contains datablock(s) I. DOI: 10.1107/S1600536814008290/su2721sup1.cif

Structure factors: contains datablock(s) I. DOI: 10.1107/S1600536814008290/su2721Isup2.hkl

Supporting information file. DOI: 10.1107/S1600536814008290/su2721Isup3.cml

CCDC reference: 997010

Additional supporting information:  crystallographic information; 3D view; checkCIF report

## Figures and Tables

**Table 1:** Hydrogen-bond geometry (Å, °)

*D*—H⋯*A*	*D*—H	H⋯*A*	*D*⋯*A*	*D*—H⋯*A*
C20—H20⋯O2^i^	0.95	2.58	3.294 (3)	132

Symmetry code: (i) 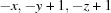.

**Fig. 1.**
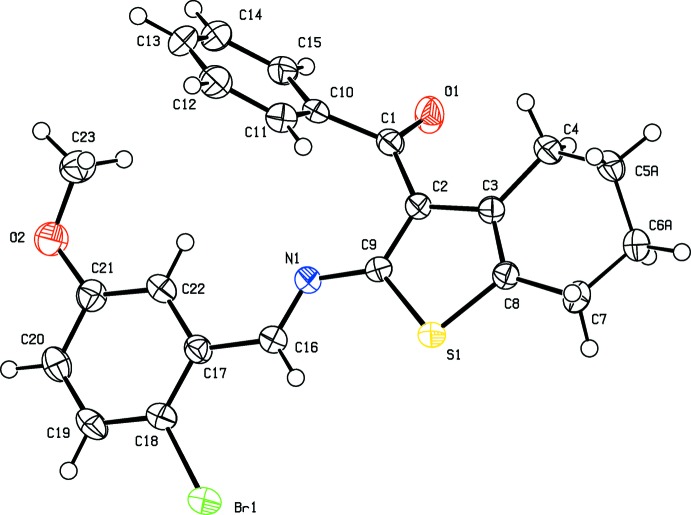
A view of the molecular structure of the title molecule, with atom labelling. Displacement ellipsoids are drawn at the 30% probability level (the minor component atoms C5B and C6B are not shown).

**Fig. 2.**
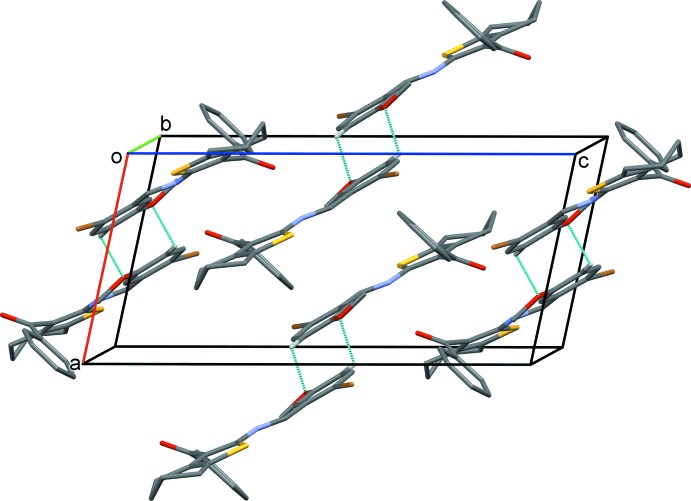
A view along the b axis of the crystal packing of the title compound. The C—H···O hydrogen bonds are shown as dashed lines (see Table 1 for details; H atoms not involved in hydrogen bonding have been omitted for clarity; the minor component atoms C5B and C6B are not shown).

## supplementary crystallographic information

### 1. Comment

2-Aminothiophene derivatives have been used in a number of applications in pesticides, dyes and pharmaceuticals (Sabnis *et al.* 1999; Puterová *et al.* 2010). Schiff base compounds show biological activities including antibacterial, antifungal, anticancer and herbicidal activities (Desai *et al.*, 2001; Karia & Parsania, 1999; Samadhiya & Halve, 2001; Singh & Dash, 1988) and have been used as starting materials in the synthesis of compounds of industrial (Aydogan *et al.*, 2001) and biological interest such as β-lactams (Taggi *et al.*, 2002). In continuation of our work on the Schiff base derivatives of 2-aminothiophenes (Kubicki *et al.*, 2012), we report herein on the crystal structure of the title compound.

In the title compound, Fig. 1, disorder was modeled for atoms C5 and C6 of the cyclohexene ring over two sites (A and B) with an occupancy ratio of 0.580 (11):0.420 (11). Both rings have half-chair conformations with puckering parameters (Cremer & Pople, 1975) Q,θ, and φ being = 0.520 (6) Å, 49.9 (4) ° and 154.8 (6) °, respectively, for ring A and being = 0.527 (8) Å, 130.1 (5) ° and 322.0 (7) °, respectively, for ring B. The dihedral angles between the mean plane of the thiophene ring and the benzene and phenyl rings are 9.2 (2) ° and 66.1 (2) °, respectively. The benzene and phenyl rings are twisted with respect to each other by 74.8 (8)°. Bond lengths are in normal ranges (Allen *et al.*, 1987).

In the crystal, molecules are linked by pairs of C-H···O hydrogen bonds forming inversion dimers (Table 1 and Fig. 2).

### 2. Experimental

To a solution of (2-amino-4,5,6,7-tetrahydro-benzo[b]thiophen-3-yl)- phenyl-methanone (200 mg, 0.79 mmol) in 10 ml of methanol an equimolar amount of 2-bromo-5-methoxybenzaldehyde (170 mg, 0.79 mmol) was added with constant stirring. The mixture was then refluxed for 6 hours and a yellow precipitate was obtained. The reaction completion was confirmed by thin layer chromatography. The precipitate was filtered and dried at room temperature overnight. Slow evaporation of a solution in CH_2_Cl_2_ gave yellow block-like crystals of the title compound.

### 3. Refinement

All H atoms were placed in calculated positions and refined as riding atoms: C—H = 0.95 - 0.99Å with U_iso_(H) = 1.5U_eq_(C-methyl) and = 1.2U_eq_(C) for other H atoms. Atoms C5 and C6, of the tetrahydrobenzothiophenyl ring, are disordered over two sites (A and B) and were refined with an occupancy ratio of 0.574 (11):0.426 (11).

### Crystal data

**Table:**

C_23_H_20_BrNO_2_S	*F*(000) = 928
*M**_r_* = 454.37	*D*_x_ = 1.508 Mg m^−^^3^
Monoclinic, *P*2_1_/*n*	Cu *K*α radiation, λ = 1.54184 Å
*a* = 8.84813 (17) Å	Cell parameters from 5917 reflections
*b* = 12.5563 (2) Å	θ = 4.3–71.4°
*c* = 18.4384 (4) Å	µ = 3.92 mm^−^^1^
β = 102.363 (2)°	*T* = 173 K
*V* = 2001.00 (7) Å^3^	Irregular, yellow
*Z* = 4	0.26 × 0.22 × 0.14 mm

### Data collection

**Table:**

Agilent Eos Gemini diffractometer	3440 reflections with *I* > 2σ(*I*)
Radiation source: Enhance (Cu) X-ray Source	*R*_int_ = 0.034
ω scans	θ_max_ = 71.3°, θ_min_ = 4.3°
Absorption correction: multi-scan (*CrysAlis PRO* and *CrysAlis RED*; Agilent, 2012)	*h* = −10→10
*T*_min_ = 0.725, *T*_max_ = 1.000	*k* = −15→13
12404 measured reflections	*l* = −22→22
3853 independent reflections	

### Refinement

**Table:**

Refinement on *F*^2^	0 restraints
Least-squares matrix: full	Hydrogen site location: inferred from neighbouring sites
*R*[*F*^2^ > 2σ(*F*^2^)] = 0.032	H-atom parameters constrained
*wR*(*F*^2^) = 0.087	*w* = 1/[σ^2^(*F*_o_^2^) + (0.0539*P*)^2^ + 0.1502*P*] where *P* = (*F*_o_^2^ + 2*F*_c_^2^)/3
*S* = 1.05	(Δ/σ)_max_ < 0.001
3853 reflections	Δρ_max_ = 0.56 e Å^−^^3^
273 parameters	Δρ_min_ = −0.31 e Å^−^^3^

### Special details

**Table:**

Geometry. All esds (except the esd in the dihedral angle between two l.s. planes) are estimated using the full covariance matrix. The cell esds are taken into account individually in the estimation of esds in distances, angles and torsion angles; correlations between esds in cell parameters are only used when they are defined by crystal symmetry. An approximate (isotropic) treatment of cell esds is used for estimating esds involving l.s. planes.

### Fractional atomic coordinates and isotropic or equivalent isotropic displacement parameters (Å^2^)

**Table:**

	*x*	*y*	*z*	*U*_iso_*/*U*_eq_	Occ. (<1)
Br1	0.09686 (3)	0.02458 (2)	0.60828 (2)	0.03755 (10)	
S1	0.37522 (6)	−0.13254 (4)	0.41239 (3)	0.02972 (12)	
O1	0.3841 (2)	0.10568 (12)	0.20797 (8)	0.0407 (4)	
O2	0.17717 (19)	0.45920 (12)	0.48309 (10)	0.0405 (4)	
N1	0.31360 (18)	0.08489 (13)	0.41394 (9)	0.0273 (3)	
C1	0.4306 (2)	0.10714 (15)	0.27533 (11)	0.0277 (4)	
C2	0.4297 (2)	0.00672 (15)	0.31824 (11)	0.0252 (4)	
C3	0.4776 (2)	−0.09409 (15)	0.29439 (10)	0.0260 (4)	
C4	0.5456 (2)	−0.11311 (16)	0.22707 (12)	0.0326 (4)	
H4AA	0.4632	−0.1074	0.1815	0.039*	0.580 (11)
H4AB	0.6246	−0.0581	0.2247	0.039*	0.580 (11)
H4BC	0.6560	−0.0922	0.2382	0.039*	0.420 (11)
H4BD	0.4906	−0.0689	0.1852	0.039*	0.420 (11)
C5A	0.6185 (8)	−0.2219 (3)	0.2307 (3)	0.0388 (14)	0.580 (11)
H5AA	0.7174	−0.2213	0.2680	0.047*	0.580 (11)
H5AB	0.6418	−0.2394	0.1819	0.047*	0.580 (11)
C6A	0.5129 (10)	−0.3069 (3)	0.2513 (3)	0.0427 (14)	0.580 (11)
H6AA	0.5568	−0.3783	0.2462	0.051*	0.580 (11)
H6AB	0.4100	−0.3028	0.2173	0.051*	0.580 (11)
C5B	0.5303 (11)	−0.2343 (5)	0.2047 (4)	0.0386 (18)	0.420 (11)
H5BA	0.4198	−0.2524	0.1865	0.046*	0.420 (11)
H5BB	0.5848	−0.2476	0.1639	0.046*	0.420 (11)
C6B	0.5977 (12)	−0.3033 (5)	0.2695 (4)	0.0411 (17)	0.420 (11)
H6BA	0.7060	−0.2820	0.2904	0.049*	0.420 (11)
H6BB	0.5974	−0.3786	0.2536	0.049*	0.420 (11)
C7	0.4959 (3)	−0.29043 (15)	0.33123 (12)	0.0353 (4)	
H7AA	0.4128	−0.3367	0.3419	0.042*	0.580 (11)
H7AB	0.5938	−0.3089	0.3662	0.042*	0.580 (11)
H7BC	0.3994	−0.3322	0.3165	0.042*	0.420 (11)
H7BD	0.5543	−0.3186	0.3793	0.042*	0.420 (11)
C8	0.4568 (2)	−0.17526 (15)	0.34010 (11)	0.0269 (4)	
C9	0.3723 (2)	−0.00049 (15)	0.38167 (11)	0.0255 (4)	
C10	0.4899 (2)	0.20856 (15)	0.31299 (10)	0.0267 (4)	
C11	0.5983 (2)	0.20984 (17)	0.38015 (12)	0.0346 (4)	
H11	0.6293	0.1451	0.4057	0.041*	
C12	0.6606 (3)	0.3059 (2)	0.40951 (14)	0.0441 (5)	
H12	0.7362	0.3069	0.4547	0.053*	
C13	0.6125 (3)	0.40071 (19)	0.37281 (15)	0.0483 (6)	
H13	0.6558	0.4664	0.3928	0.058*	
C14	0.5021 (3)	0.39969 (18)	0.30749 (14)	0.0444 (5)	
H14	0.4674	0.4647	0.2832	0.053*	
C15	0.4416 (3)	0.30354 (16)	0.27719 (12)	0.0345 (4)	
H15	0.3668	0.3029	0.2318	0.041*	
C16	0.2623 (2)	0.07449 (15)	0.47319 (11)	0.0277 (4)	
H16	0.2647	0.0067	0.4964	0.033*	
C17	0.1995 (2)	0.16678 (16)	0.50565 (11)	0.0281 (4)	
C18	0.1202 (2)	0.15806 (16)	0.56306 (11)	0.0299 (4)	
C19	0.0562 (2)	0.24697 (18)	0.58977 (12)	0.0348 (4)	
H19	−0.0009	0.2392	0.6276	0.042*	
C20	0.0754 (2)	0.34625 (17)	0.56152 (12)	0.0358 (5)	
H20	0.0319	0.4071	0.5799	0.043*	
C21	0.1592 (2)	0.35725 (16)	0.50552 (12)	0.0319 (4)	
C22	0.2189 (2)	0.26847 (16)	0.47765 (11)	0.0299 (4)	
H22	0.2739	0.2763	0.4390	0.036*	
C23	0.2887 (3)	0.47572 (17)	0.43904 (14)	0.0413 (5)	
H23A	0.2536	0.4414	0.3906	0.062*	
H23B	0.3879	0.4449	0.4642	0.062*	
H23C	0.3015	0.5523	0.4320	0.062*	

### Atomic displacement parameters (Å^2^)

**Table:**

	*U*^11^	*U*^22^	*U*^33^	*U*^12^	*U*^13^	*U*^23^
Br1	0.04351 (15)	0.03807 (15)	0.03571 (15)	−0.00469 (8)	0.01879 (10)	0.00195 (8)
S1	0.0388 (3)	0.0250 (2)	0.0288 (2)	−0.00344 (17)	0.0148 (2)	0.00060 (17)
O1	0.0647 (10)	0.0314 (7)	0.0247 (7)	0.0017 (7)	0.0063 (7)	−0.0007 (6)
O2	0.0448 (9)	0.0312 (7)	0.0485 (9)	0.0077 (6)	0.0168 (7)	0.0049 (7)
N1	0.0286 (8)	0.0273 (8)	0.0282 (8)	−0.0024 (6)	0.0107 (6)	−0.0035 (6)
C1	0.0306 (9)	0.0271 (9)	0.0278 (10)	0.0021 (7)	0.0116 (7)	0.0006 (7)
C2	0.0252 (9)	0.0253 (9)	0.0259 (9)	−0.0015 (7)	0.0075 (7)	−0.0021 (7)
C3	0.0256 (8)	0.0259 (9)	0.0270 (9)	−0.0015 (7)	0.0070 (7)	−0.0027 (7)
C4	0.0370 (10)	0.0313 (10)	0.0336 (11)	0.0019 (8)	0.0167 (8)	−0.0013 (8)
C5A	0.046 (3)	0.031 (2)	0.046 (3)	0.0047 (19)	0.027 (3)	−0.0045 (17)
C6A	0.058 (4)	0.028 (2)	0.051 (3)	−0.003 (2)	0.030 (3)	−0.0095 (18)
C5B	0.044 (4)	0.038 (3)	0.037 (3)	−0.001 (3)	0.016 (3)	−0.010 (2)
C6B	0.047 (4)	0.033 (3)	0.047 (4)	0.005 (3)	0.017 (3)	−0.011 (2)
C7	0.0453 (11)	0.0235 (10)	0.0386 (11)	−0.0014 (8)	0.0125 (9)	−0.0031 (8)
C8	0.0262 (9)	0.0260 (9)	0.0291 (10)	−0.0018 (7)	0.0073 (7)	−0.0033 (7)
C9	0.0272 (9)	0.0231 (8)	0.0278 (9)	−0.0023 (7)	0.0092 (7)	0.0002 (7)
C10	0.0308 (9)	0.0267 (9)	0.0267 (9)	−0.0021 (7)	0.0154 (7)	−0.0011 (7)
C11	0.0352 (10)	0.0351 (11)	0.0354 (11)	−0.0033 (8)	0.0119 (8)	−0.0009 (8)
C12	0.0424 (12)	0.0498 (14)	0.0425 (13)	−0.0135 (10)	0.0144 (10)	−0.0113 (10)
C13	0.0623 (15)	0.0338 (12)	0.0562 (15)	−0.0214 (11)	0.0295 (12)	−0.0137 (11)
C14	0.0671 (15)	0.0253 (10)	0.0479 (13)	−0.0045 (10)	0.0279 (12)	0.0012 (9)
C15	0.0463 (12)	0.0289 (10)	0.0323 (11)	−0.0002 (8)	0.0169 (9)	0.0023 (8)
C16	0.0282 (9)	0.0281 (9)	0.0279 (9)	−0.0009 (7)	0.0086 (7)	−0.0006 (7)
C17	0.0256 (9)	0.0311 (10)	0.0288 (10)	0.0003 (7)	0.0085 (7)	−0.0031 (8)
C18	0.0289 (9)	0.0342 (10)	0.0286 (10)	−0.0008 (7)	0.0107 (8)	0.0012 (8)
C19	0.0290 (9)	0.0471 (12)	0.0318 (10)	0.0034 (8)	0.0140 (8)	−0.0043 (9)
C20	0.0329 (10)	0.0377 (11)	0.0387 (11)	0.0095 (8)	0.0123 (9)	−0.0047 (9)
C21	0.0297 (9)	0.0304 (10)	0.0352 (11)	0.0042 (7)	0.0061 (8)	0.0004 (8)
C22	0.0291 (9)	0.0356 (10)	0.0270 (9)	0.0015 (7)	0.0105 (8)	−0.0007 (8)
C23	0.0443 (12)	0.0361 (12)	0.0447 (13)	−0.0001 (9)	0.0121 (10)	0.0061 (9)

### Geometric parameters (Å, º)

**Table:**

Br1—C18	1.903 (2)	C6B—C7	1.603 (7)
S1—C8	1.7314 (19)	C7—H7AA	0.9900
S1—C9	1.7506 (19)	C7—H7AB	0.9900
O1—C1	1.222 (2)	C7—H7BC	0.9900
O2—C21	1.365 (3)	C7—H7BD	0.9900
O2—C23	1.421 (3)	C7—C8	1.504 (3)
N1—C9	1.380 (3)	C10—C11	1.395 (3)
N1—C16	1.277 (2)	C10—C15	1.386 (3)
C1—C2	1.489 (3)	C11—H11	0.9500
C1—C10	1.491 (3)	C11—C12	1.387 (3)
C2—C3	1.433 (3)	C12—H12	0.9500
C2—C9	1.374 (3)	C12—C13	1.391 (4)
C3—C4	1.510 (3)	C13—H13	0.9500
C3—C8	1.360 (3)	C13—C14	1.379 (4)
C4—H4AA	0.9900	C14—H14	0.9500
C4—H4AB	0.9900	C14—C15	1.389 (3)
C4—H4BC	0.9900	C15—H15	0.9500
C4—H4BD	0.9900	C16—H16	0.9500
C4—C5A	1.506 (4)	C16—C17	1.467 (3)
C4—C5B	1.575 (6)	C17—C18	1.394 (3)
C5A—H5AA	0.9900	C17—C22	1.402 (3)
C5A—H5AB	0.9900	C18—C19	1.389 (3)
C5A—C6A	1.519 (8)	C19—H19	0.9500
C6A—H6AA	0.9900	C19—C20	1.375 (3)
C6A—H6AB	0.9900	C20—H20	0.9500
C6A—C7	1.526 (5)	C20—C21	1.401 (3)
C5B—H5BA	0.9900	C21—C22	1.379 (3)
C5B—H5BB	0.9900	C22—H22	0.9500
C5B—C6B	1.494 (12)	C23—H23A	0.9800
C6B—H6BA	0.9900	C23—H23B	0.9800
C6B—H6BB	0.9900	C23—H23C	0.9800
			
C8—S1—C9	91.38 (9)	C8—C7—H7AA	110.1
C21—O2—C23	116.75 (16)	C8—C7—H7AB	110.1
C16—N1—C9	121.68 (17)	C8—C7—H7BC	109.5
O1—C1—C2	119.22 (18)	C8—C7—H7BD	109.5
O1—C1—C10	119.64 (18)	C3—C8—S1	112.26 (14)
C2—C1—C10	121.13 (17)	C3—C8—C7	126.05 (18)
C3—C2—C1	123.31 (17)	C7—C8—S1	121.68 (15)
C9—C2—C1	123.55 (17)	N1—C9—S1	125.24 (14)
C9—C2—C3	112.97 (17)	C2—C9—S1	110.80 (15)
C2—C3—C4	126.00 (17)	C2—C9—N1	123.88 (18)
C8—C3—C2	112.57 (17)	C11—C10—C1	122.00 (18)
C8—C3—C4	121.43 (17)	C15—C10—C1	118.12 (18)
C3—C4—H4AA	109.6	C15—C10—C11	119.77 (19)
C3—C4—H4AB	109.6	C10—C11—H11	120.1
C3—C4—H4BC	109.7	C12—C11—C10	119.8 (2)
C3—C4—H4BD	109.7	C12—C11—H11	120.1
C3—C4—C5B	110.0 (3)	C11—C12—H12	120.0
H4AA—C4—H4AB	108.1	C11—C12—C13	120.0 (2)
H4BC—C4—H4BD	108.2	C13—C12—H12	120.0
C5A—C4—C3	110.4 (2)	C12—C13—H13	119.9
C5A—C4—H4AA	109.6	C14—C13—C12	120.2 (2)
C5A—C4—H4AB	109.6	C14—C13—H13	119.9
C5B—C4—H4BC	109.7	C13—C14—H14	120.0
C5B—C4—H4BD	109.7	C13—C14—C15	120.0 (2)
C4—C5A—H5AA	109.3	C15—C14—H14	120.0
C4—C5A—H5AB	109.3	C10—C15—C14	120.2 (2)
C4—C5A—C6A	111.6 (5)	C10—C15—H15	119.9
H5AA—C5A—H5AB	108.0	C14—C15—H15	119.9
C6A—C5A—H5AA	109.3	N1—C16—H16	119.9
C6A—C5A—H5AB	109.3	N1—C16—C17	120.24 (18)
C5A—C6A—H6AA	109.7	C17—C16—H16	119.9
C5A—C6A—H6AB	109.7	C18—C17—C16	122.95 (18)
C5A—C6A—C7	109.8 (5)	C18—C17—C22	118.20 (18)
H6AA—C6A—H6AB	108.2	C22—C17—C16	118.85 (17)
C7—C6A—H6AA	109.7	C17—C18—Br1	121.43 (15)
C7—C6A—H6AB	109.7	C19—C18—Br1	117.50 (15)
C4—C5B—H5BA	109.5	C19—C18—C17	121.06 (19)
C4—C5B—H5BB	109.5	C18—C19—H19	120.0
H5BA—C5B—H5BB	108.1	C20—C19—C18	120.08 (19)
C6B—C5B—C4	110.7 (6)	C20—C19—H19	120.0
C6B—C5B—H5BA	109.5	C19—C20—H20	120.1
C6B—C5B—H5BB	109.5	C19—C20—C21	119.75 (19)
C5B—C6B—H6BA	109.9	C21—C20—H20	120.1
C5B—C6B—H6BB	109.9	O2—C21—C20	115.47 (18)
C5B—C6B—C7	108.8 (6)	O2—C21—C22	124.50 (19)
H6BA—C6B—H6BB	108.3	C22—C21—C20	120.02 (19)
C7—C6B—H6BA	109.9	C17—C22—H22	119.6
C7—C6B—H6BB	109.9	C21—C22—C17	120.81 (18)
C6A—C7—H7AA	110.1	C21—C22—H22	119.6
C6A—C7—H7AB	110.1	O2—C23—H23A	109.5
C6B—C7—H7BC	109.5	O2—C23—H23B	109.5
C6B—C7—H7BD	109.5	O2—C23—H23C	109.5
H7AA—C7—H7AB	108.4	H23A—C23—H23B	109.5
H7BC—C7—H7BD	108.1	H23A—C23—H23C	109.5
C8—C7—C6A	107.9 (2)	H23B—C23—H23C	109.5
C8—C7—C6B	110.6 (3)		
			
Br1—C18—C19—C20	177.08 (17)	C5B—C6B—C7—C8	−43.3 (8)
O1—C1—C2—C3	42.7 (3)	C6B—C7—C8—S1	−169.4 (4)
O1—C1—C2—C9	−132.2 (2)	C6B—C7—C8—C3	11.4 (5)
O1—C1—C10—C11	−153.30 (19)	C8—S1—C9—N1	177.94 (17)
O1—C1—C10—C15	22.8 (3)	C8—S1—C9—C2	0.96 (15)
O2—C21—C22—C17	177.79 (19)	C8—C3—C4—C5A	−13.9 (4)
N1—C16—C17—C18	169.70 (19)	C8—C3—C4—C5B	19.7 (5)
N1—C16—C17—C22	−9.9 (3)	C9—S1—C8—C3	−1.23 (15)
C1—C2—C3—C4	4.7 (3)	C9—S1—C8—C7	179.49 (17)
C1—C2—C3—C8	−175.86 (17)	C9—N1—C16—C17	−179.50 (17)
C1—C2—C9—S1	174.92 (15)	C9—C2—C3—C4	−179.91 (18)
C1—C2—C9—N1	−2.1 (3)	C9—C2—C3—C8	−0.4 (2)
C1—C10—C11—C12	174.01 (19)	C10—C1—C2—C3	−136.11 (19)
C1—C10—C15—C14	−175.38 (19)	C10—C1—C2—C9	48.9 (3)
C2—C1—C10—C11	25.5 (3)	C10—C11—C12—C13	1.4 (3)
C2—C1—C10—C15	−158.38 (18)	C11—C10—C15—C14	0.8 (3)
C2—C3—C4—C5A	165.5 (3)	C11—C12—C13—C14	0.5 (4)
C2—C3—C4—C5B	−160.9 (4)	C12—C13—C14—C15	−1.7 (4)
C2—C3—C8—S1	1.2 (2)	C13—C14—C15—C10	1.1 (3)
C2—C3—C8—C7	−179.59 (18)	C15—C10—C11—C12	−2.0 (3)
C3—C2—C9—S1	−0.5 (2)	C16—N1—C9—S1	3.8 (3)
C3—C2—C9—N1	−177.51 (17)	C16—N1—C9—C2	−179.59 (19)
C3—C4—C5A—C6A	46.7 (7)	C16—C17—C18—Br1	3.6 (3)
C3—C4—C5B—C6B	−53.2 (8)	C16—C17—C18—C19	−176.87 (19)
C4—C3—C8—S1	−179.33 (15)	C16—C17—C22—C21	178.74 (18)
C4—C3—C8—C7	−0.1 (3)	C17—C18—C19—C20	−2.4 (3)
C4—C5A—C6A—C7	−67.6 (8)	C18—C17—C22—C21	−0.9 (3)
C4—C5B—C6B—C7	65.5 (9)	C18—C19—C20—C21	0.2 (3)
C5A—C4—C5B—C6B	43.5 (7)	C19—C20—C21—O2	−177.5 (2)
C5A—C6A—C7—C6B	−51.1 (7)	C19—C20—C21—C22	1.6 (3)
C5A—C6A—C7—C8	49.3 (7)	C20—C21—C22—C17	−1.3 (3)
C6A—C7—C8—S1	161.2 (4)	C22—C17—C18—Br1	−176.77 (14)
C6A—C7—C8—C3	−17.9 (4)	C22—C17—C18—C19	2.7 (3)
C5B—C4—C5A—C6A	−48.4 (6)	C23—O2—C21—C20	166.49 (19)
C5B—C6B—C7—C6A	46.2 (7)	C23—O2—C21—C22	−12.6 (3)

### Hydrogen-bond geometry (Å, º)

**Table:**

*D*—H···*A*	*D*—H	H···*A*	*D*···*A*	*D*—H···*A*
C20—H20···O2^i^	0.95	2.58	3.294 (3)	132

Symmetry code: (i) −*x*, −*y*+1, −*z*+1.
